# Detection, Monitoring, and Mitigation of Drug-Induced Nephrotoxicity: A Pragmatic Approach

**DOI:** 10.1007/s43441-023-00599-x

**Published:** 2023-12-18

**Authors:** Nicola Antognini, Ronald Portman, Victor Dong, Nicholas J. Webb, Deepa H. Chand

**Affiliations:** 1 Novartis Pharma AG, Basel, Switzerland; 2 Novartis Pharmaceuticals, East Hanover, NJ USA; 3 University of Illinois College of Medicine-Peoria, Children’s Hospital of Illinois, Peoria, IL USA

**Keywords:** Acute kidney injury, Chronic kidney disease, Biomarkers, Creatinine, Drug-induced nephrotoxicity, Glomerular filtration rate, Nephrotoxicity, Monitoring, Drug development

## Abstract

The kidneys play a pivotal role in elimination of most drugs; therefore, a comprehensive understanding of renal physiology and pathology is important for those involved in drug development. High filtration capacity and metabolic activity make the kidneys vulnerable to drug-induced nephrotoxicity (DIN). Acute DIN may manifest on a background of renal impairment that has resulted from underlying disease, previously administered nephrotoxic medications, congenital renal abnormalities, or the natural aging process. The ability of the kidneys to compensate for DIN depends on the degree of pre-insult renal function. Therefore, it can be difficult to identify. The discovery and development of novel biomarkers that can diagnose kidney damage earlier and more accurately than current clinical measures and may be effective in detecting DIN. The goal of this manuscript is to provide a pragmatic and evidence-based supportive guidance for the early identification and management of DIN during the drug development process for clinical trial participants of all ages. The overall objective is to minimize the impact of DIN on kidney function and to collect renal safety data enabling risk analysis and mitigation.

## Introduction

The kidneys are complex organs with many functions including blood cleansing through glomerular filtration, tubular reabsorption, and tubular secretion.

Glomerular ultrafiltration of plasma begins in the 9th week of gestation. During gestation, glomerular filtration rate (GFR) increases in parallel with gestational age until a large increase occurs at the time of completion of nephrogenesis, which is achieved at approximately 35–36 weeks of gestation [1]. Of note, certain maternal medications can influence renal development in utero. One example of this is the use of non-steroidal anti-inflammatory drugs after 20 weeks of gestation can result in prostaglandin receptor blockade as the mechanism for reduced renal perfusion with resultant oligohydramnios, leading to neonatal renal impairment [2].

Most drugs and their metabolites are eliminated in the urine by glomerular filtration and/or tubular secretion. The ultrafiltrate formed in the glomeruli is modified through tubular transport, mostly in the proximal tubule. In the context of nephrotoxicity, tubular cell uptake of potentially nephrotoxic drugs occurs via the apical pathways via endocytosis/pinocytosis and other passive or active transport. Alternatively, uptake can occur through the basolateral membranes of the proximal tubules via the peritubular capillaries [3].

The kidneys are especially predisposed and vulnerable to the toxic effects of drugs and their metabolites as they can reach high levels of local exposure in renal tubules and interstitium, which could lead to DIN [4]. For example, some drugs (e.g., aminoglycosides, cyclosporine, cisplatin, amphotericin B) have direct proximal tubular cell toxicity, and their use is associated with an increased risk of kidney damage. Therefore, drug-induced renal effects are an important consideration in drug development that require prediction, vigilance, early detection, and adequate risk mitigation.

While AKI often resolves if the underlying etiology is corrected, it may also lead to chronic, irreversible histologic changes within the kidneys. Signs and symptoms of nephrotoxicity can vary and span a broad spectrum, reflecting damage to different nephron segments, potentially resulting in proteinuria, hematuria, oliguria, dysregulated acid–base balance, and/or electrolyte abnormalities. Ultimately, DIN can present as either acute kidney injury (AKI) or chronic kidney disease (CKD).

The kidneys have significant functional reserve; and while their regenerative capacity is limited, they can adapt, as exemplified by the ability to donate one kidney, and can compensate for mild degrees of renal impairment by increasing function in other segments of a nephron (i.e., hypertrophy), or by increasing function of other healthy nephrons (i.e., recruitment). The kidneys increasingly depend on functional reserve capacity with increasing age. Each DIN event further decreases the reserve capacity and thus, potentially leads to an earlier onset of functional renal impairment, even in response to mild injury that may be difficult to detect with clinical testing.

In this manuscript, the authors provide a comprehensive overview of DIN, including a classification schema to allow for appropriate identification and mitigation of DIN based on patient population, intrinsic pharmaceutical agent composition, pharmacokinetic properties, and results of preclinical studies. Further, monitoring and mitigation strategies are presented, including caveats pertaining to special populations such as children and cancer patients.

## Diagnosis and Classification of AKI and CKD

AKI can span a spectrum from mild forms to more advanced injury necessitating renal replacement therapy (RRT). Clinically, AKI is characterized by a reduction in renal function resulting in a failure to maintain fluid, electrolyte, and/or acid–base homeostasis. The acute loss in renal function may manifest as accumulation of end products of nitrogen metabolism, oliguria, metabolic acidosis, and/or electrolyte abnormalities [5].

In 2012, the KDIGO working group combined the RIFLE [6] and AKIN [7] classifications to establish one classification of AKI for practice, research, and public health (Table 1) [8]. AKI can be due to pre-renal, renal, or post-renal etiologies. AKI often has an abrupt onset and is in principle reversible. Therefore, early identification and prompt management are critical. Once the diagnosis of AKI has been confirmed or suspected, the patient should receive immediate adequate specialized treatment to minimize the potential to become chronic and irreversible.

**Table 1.** AKI—Diagnostic Criteria, Staging, and Classification.

	RIFLE^a^	AKIN^a^	KDIGO^a^
Diagnostic criteria^b^			
		Increase in serum creatinine of ≥ 0.3 mg/dL or ≥ 50% within 48 h OR Urine output of < 0.5 mL/kg/hour for > 6 h	Increase in serum creatinine of ≥ 0.3 mg/dL within 48 h or ≥ 50% within 7 days OR Urine output of < 0.5 mL/kg/hour for > 6 h
Staging criteria			
Risk (RIFLE) or stage 1 (AKIN/KDIGO)	Increase in serum creatinine to 1.5 times baseline OR Urine output of < 0.5 mL/kg/hour for 6 to 12 h	Increase in serum creatinine of ≥ 0.3 mg/dL or to 150 to 200% baseline OR Urine output of < 0.5 mL/kg/hour for 6 to 12 h	Increase in serum creatinine of ≥ 0.3 mg/dL or 1.5 to 1.9 times baseline OR Urine output of < 0.5 mL/kg/hour for 6 to 12 h
Injury (RIFLE) or stage 2 (AKIN/KDIGO)	Increase in serum creatinine to 2 times baseline OR Urine output of < 0.5 mL/kg/hour for 12 to 24 h	Increase in serum creatinine to 200 to 300% baseline OR Urine output of < 0.5 mL/kg/hour for 12 to 24 h	Increase in serum creatinine to 2 to 2.9 times baseline OR Urine output of < 0.5 mL/kg/hour for 12 to 24 h
Failure (RIFLE) or stage 3 (AKIN/KDIGO)	Increase in serum creatinine to 3 times baseline OR Increase in serum creatinine by > 0.5 mg/dL to > 4 mg/dL OR Urine output of < 0.3 mL/kg/hour for > 24 h or anuria for > 12 h OR Initiation of kidney replacement therapy	Increase in serum creatinine to > 300% baseline OR Increase in serum creatinine by > 0.5 mg/dL to ≥ 4 mg/dL OR Urine output of < 0.3 mL/kg/hour for > 24 h or anuria for > 12 h OR Initiation of kidney replacement therapy	Increase in serum creatinine to ≥ 3 times baseline OR Increase in serum creatinine of ≥ 0.3 mg/dL to ≥ 4 mg/dL¶ OR Urine output of < 0.3 mL/kg/hour for ≥ 24 h or anuria for ≥ 12 h OR Initiation of kidney replacement therapy
Loss (RIFLE)	Need for kidney replacement therapy for > 4 weeks		
End stage (RIFLE)	Need for kidney replacement therapy for > 3 months		
Classification			
	Mechanism of toxicity	Examples	
Pre-renal	Indirect, by causing renal ischemia or decreased renal perfusion	Dehydration	
		Hemorrhage	
		Sepsis or septicemia	
		Heart failure	
		Liver failure	
		Major trauma, e.g., burn wounds	
		Major surgery, e.g., CABG (coronary bypass surgery)	
Renal	Direct nephrotoxic effect/kidney damage	Nephrotoxic drugs, e.g., aminoglycosides	
		Chronic drug use, e.g., NSAIDs	
		Drug abuse or overdose	
		Radiocontrast media	
		Acute infection (pyelonephritis)	
		Immunologic injury (immune complex disease, serum sickness, hemolytic uremic syndrome, idiosyncratic drug reactions, glomerulonephritis, rhabdomyolysis, etc.)	
		Tumor lysis syndrome	
Post-renal	Indirect, by preventing urinary outflow	Prostatism and prostatitis	
		Bladder or pelvic tumors	
		Renal calculi	
		Congenital urinary tract obstruction	
		Anticholinergics	

^a^*RIFLE* risk, injury, failure, loss, *AKIN* Acute Kidney Injury Network, *KDIGO* kidney disease: improving global outcomes

^b^AKIN and KDIGO provided both diagnostic and staging criteria. RIFLE provided a graded definition of AKI that is implicit in the staging criteria

In patients < 18 years, stage 3 AKI is also defined by KDIGO as a decrease in estimated glomerular filtration rate (eGFR) to < 35 mL/min/1.73 m^2^

Urine output is one of the most easily measured parameters and, as such, is an important indicator of renal function used in the modern classification of AKI [6]. It may be a more sensitive index of changes in renal hemodynamics than biochemical markers of solute clearance. However, it has insufficient sensitivity and specificity as a marker of renal function in patients with AKI due to impaired free water and solute excretion. In patients with severe AKI, urine output could be normal or even elevated due to tubulo-interstitial injury [9].

Laboratory assessment of renal function even in the presence of considerable renal damage, serum creatinine (SCr), blood urea nitrogen (BUN), and estimated glomerular filtration rate (eGFR) may remain stable, as seen in the slow, progressive, and asymptomatic renal function decline that commonly occurs in the elderly as the total number of functioning nephrons gradually decreases with age. A disadvantage is that changes in SCr occur no earlier than 24 to 48 h after kidney damage, which delays the diagnosis of DIN, increasing the risk of irreversible kidney damage. Further, muscle mass also decreases with age and thus only small increases in creatinine may underestimate the magnitude of renal injury. A biomarker representing the number of functional nephrons is not yet available.

Chronic kidney disease is defined as either kidney damage or decreased eGFR for at least 3 months leading to long-term, irreversible renal damage and is staged based on severity (Table 2). Kidneys with chronic damage are often more susceptible to acute injury. Early or mild degrees of renal impairment are seldom associated with significant long-term serum creatinine increases or clinical manifestations; hence measurement of creatinine may not be sufficient for identifying Stage 1 and Stage 2 CKD. In such cases, the presence of one or more of the markers listed in Table 2 may help in identifying early CKD.

**Table 2.** CKD Stages and Markers.

Stage^a^	Description	eGFR (mL/min/1.73 m^2^)
1	Abnormal blood, urine, or imaging studies with normal or slightly decreased GFR	≥ 90
2	Abnormal blood, urine, or imaging studies with mildly decreased GFR	60–89
3a	Moderately decreased GFR	45–59
3b	Moderately decreased GFR	30–44
4	Severely decreased GFR	15–29
5	End-stage kidney failure	< 15
CKD markers		
Albuminuria (albumin excretion > 30 mg/24 h or albumin:creatinine ratio (ACR) > 30 mg/g [> 3 mg/mmol])		
Urine sediment abnormalities		
Electrolyte and other abnormalities due to tubular disorders		
Structural abnormalities detected by imaging		

^a^Stages of CKD in children and adults age > 2 years.

A measured GFR is considered the best marker of kidney function; however, pragmatically, eGFR is useful to determine the prognosis of kidney disease, anticipate complications, and adapt drug dosage. Glomerular filtration is the main mechanism responsible for drug elimination; therefore, drug renal clearance will depend mainly on the rate of glomerular filtration. For infants, this rate is a function of gestational and post-natal ages. Thus, even outside the context of AKI, eGFR is important for correct drug dosing. Pharmacological adaptation (such as reduced dosage or longer administration intervals) is then essential to limit drug toxicity in patient with renal dysfunction. While glomerular filtration is often responsible for drug elimination, tubular excretion may also contribute and markers including serum electrolytes should be monitored. With a decline in GFR, markers such as urine output, SCr, BUN, and cystatin C change significantly, and each marker has unique advantages and some limitations for detecting changes in glomerular filtration (Tables 3, 4).

**Table 3.** Lab or at or y Assessment of Renal Function.

Test	Description	Advantages	Limitations	Note
GFR				
Inulin renal clearance Iohexol plasma clearance	Inulin is a polymer of fructose (5200 Daltons), is found in Jerusalem artichokes, dahlias, and chicory and was first used for measuring GFR in 1951. It is considered the gold standard to precisely measure GFR	They both have all the properties of an ideal marker. They are freely filtered by the glomerulus, are not secreted, or reabsorbed in the tubules, and are not synthetized or metabolized by the kidney	Inulin use is limited because purified inulin is expensive and difficult to measure and measuring glomerular filtration rate in this way is time consuming for both patients and clinicians	In specific situations where the accurate assessment is imperative to know an actual GFR to determine accurate dosing or in situations where even small changes in GFR are significant
	Iohexol, a non-ionic, non-radioactive X-ray contrast medium (821 Daltons), has been recognized as a marker for accurate measurement of GFR since the 1990s	Iohexol does not require urine collection but plasma samples are needed. It is an excellent alternative to inulin and radioisotopes for clearance studies. Iohexol approach is faster and less cumbersome than inulin	Iohexol may have mild nephrotoxicity	
Serum biomarkers				
Creatinine (SCr)	A low-molecular-weight (113 Da) product of nonenzymatic conversion of creatine to phosphocreatinine. It is usually produced at a fairly constant rate by the body and released from muscles at a constant rate, resulting in a stable plasma concentration	It fulfills almost all of the requirements for a marker substance; it is freely filterable, not metabolized, and not reabsorbed once filtered. It has an inverse, but nonlinear, relationship with GFR such that even a minor change in SCr reflects substantial decline in GFR due to compensatory redistribution of workload toward nephrons that are less severely affected by injury	SCr is influenced by body size, sex, and muscle mass As much as 10–40% of an estimated creatinine is cleared by tubular secretion [11] and SCr requires time to accumulate in the blood even after a significant fall in GFR	It is part of chemistry panels as simple blood test SCr remains the most common and validated assessment of renal function and as intra-individual serum creatinine variability is low, an individual’s SCr change from baseline is a sensitive marker of altered renal function [12]
Cystatin C (SCysC)	Low-molecular-weight (13,000 Da) endogenous cysteine proteinase inhibitor that is synthesized at a relatively constant rate and released into the plasma by all nucleated cells in the body. It is freely (> 99%) filtered by the glomerulus and then nearly completely metabolized by proximal tubular cells. It is not secreted by tubules; thus, no or only very little amount can be detected in urine [13–15]	It is more sensitive than SCr [16] and it is not influenced by muscle mass, age, sex or diet. Elevated urinary levels of SCysC may reflect tubular damage independent from GFR	Limited availability of equipment for its determination, and high cost of assays [9]. Influenced by active immune activation giving false results	Can be used in phase I and II for early detection of nephrotoxicity It is part of one of the seven urinary biomarkers accepted by FDA/EMA [17] to monitor drug-induced kidney injury in preclinical studies and on a case-by-case basis in clinical trials
Blood urea nitrogen (BUN)	A low-molecular-weight (60 Da) product of protein catabolism that inversely correlates with GFR. It is freely filtered in the glomerulus, and as much as 40–50% of filtered urea can be reabsorbed by the tubules	It gives an estimation of the GFR, and it is available as part of chemistry panels as simple blood test	Because the liver is the single organ that has enzymes for urea synthesis, acute or chronic liver diseases could lead to near-normal values for urea independent of kidney function Urea requires time to accumulate and thus does not represent real-time changes in GFR The production of urea is not constant and varies with protein intake, liver function, and catabolic rate. In addition, urea can be reabsorbed once filtered into the kidney, and this reabsorption increases in conditions with low urine flow, such as volume depletion. Volume depletion is one cause of a high (> 15:1) BUN-to-creatinine ratio in plasma	All these limitations make BUN not suitable as GFR marker in clinical practice, but it remains a valuable tool to differentiate pre-renal AKI [9]
Urine biomarkers				
24-h urine protein collection	For quantitative measure of protein excretion Urine protein excretion over 24 h in healthy adults is between 20–150 mg The appearance of significant amount of protein in urine is inherent to nearly all progressive renal diseases. This presence of small amounts of albumin in the urine is the condition called microalbuminuria (ACR 30–300 mg/g)	It is still considered as a gold standard, but it is less used; need to standardize by including assessment of urine creatinine	24-h urine collection are required This method is prone to errors	Historically, 24-h urine collections have been performed at baseline and periodically during the study to measure urine volume and assess renal function. However, 24-h GFR measurement may not provide additional clinically significant value over serum creatinine-based eGFR in the assessment of DIN. Therefore, they are not routinely used for the assessment of renal function
Proteinuria (albumin)	As kidney damage progresses and the amount of albumin in the urine increases, the name of the condition changes from microalbuminuria to albuminuria (macroalbuminuria ACR > 300), otherwise known as proteinuria, associated with progressive decline in glomerular filtration rate	One of the initial investigations for proteinuria is a dipstick urinalysis as this is relatively low cost and easily performed	The dipstick analysis is only sensitive to albumin and influenced by urine concentration. False positive can occur with alkaline urine	Estimation of proteinuria helps to follow the progress of renal disease and to assess the response to therapy Any finding of dipstick proteinuria should be confirmed by a PCR (protein to creatinine ratio), preferably on a first morning void to exclude situations of orthostatic proteinuria
Glomerular hematuria	It may be microscopic or gross, asymptomatic, or symptomatic and may be associated with other urinary tract abnormalities. Microscopic hematuria is defined as the presence of at least five red blood cells/high power field (HPF). Hematuria can occur as a result of structural alterations due to an injury, infection, or a mass. Be sure to inquire in females if menstruation is occurring at the time of sample collection	Urinalysis is the initial and most useful test to perform Urine dipstick is widely available and can be performed quickly	Urine dipstick can give false-positive or false-negative results Myoglobinuria or hemoglobinuria may result in positive dipstick readings	In the setting of a positive urine dipstick for blood, a microscopic examination should be performed to evaluate for the presence of red blood cells (RBCs) or RBC casts Dysmorphic RBCs are found in the urine of patients with glomerular etiology, whereas isomorphic RBCs characterize non glomerular or urological hematuria [18]. A urine specimen with significant white blood cells (WBCs) and positive nitrites and leukocyte esterase may represent urinary tract infection, a likely cause of hematuria
Estimated GFR				
Chronic Kidney Disease Epidemiology Collaboration (CKD-EPI) equation [19]	Estimates GFR from serum creatinine, age and sex	Urine collection not required. Compared to SCr, eGFR provides a more reliable assessment of absolute renal function due to its ability to account for key differences among individuals which may alter the relationship between SCr and GFR, particularly in more severe degrees of renal impairment More accurate than the MDRD Study equation, particularly in people with higher levels of GFR	Like all other creatinine-based estimation equations, it suffers from physiologic limitations of creatinine as a filtration marker. Formula developed with patients with CKD; not effective for changes with normal renal function	The recommended formula is the creatinine-based eGFR equation CKD-EPI 2021, which does not include, compared to the 2009 CKD-EPI equation, a race coefficient, as it is considered a social and not a biological construct
Cockcroft-Gault equation	Estimates creatinine clearance from SCr, body weight and sex	Urine collection not required	Formula developed in people with normal GFR, not applicable to people with CKD. May overestimate GFR in overweight or obese subjects	No longer recommended because it has not been expressed using standardized creatinine values and underestimates the GFR in healthy people with GFR > 60 mL/min. The CKD-EPI equation is preferred because it circumvents this problem
Modification of Diet in Renal Disease (MDRD) equation [20]	Estimates GFR from age, SCr, BUN, albumin and sex	Urine collection not required. Developed in patients with CKD Formula without BUN and albumin provides more accurate results	GFR can be underestimated in patients with normal GFR and early CKD	The CKD-EPI equation is replacing the MDRD
The modified bedside Schwartz formula [21–23]	The most frequently used creatinine-based eGFR equation in children	Unlike the original bedside Schwartz, the revised bedside equation does not consider differences in sex The original Schwartz equation was overestimating the GFR compared with the measured GFR due to changes in creatinine measurement and calibration over time	The equation must be applied to a stable creatinine value, and is inaccurate when serum creatinine is rapidly changing The equation is not useful in children with reduced muscle mass, amputations, cachexia, or those taking creatine supplements	It is a clinical standard for estimating pediatric GFR and is the most feasible for routine safety monitoring in clinical trials and in clinical practice
Creatinine-based estimating equations are not recommended for use with subjects with extremes in muscle mass and diet. This includes, but is not limited to, individuals who are amputees, paraplegics, or bodybuilders, or have obesity; patients who have a muscle-wasting disease or a neuromuscular disorder; and those suffering from malnutrition, eating a vegetarian or low-meat diet, or taking creatine dietary supplements It is important to note that these equations are appropriate only for adults; in children, GFR estimation must take into account the normal age-related developmental stage and body measurement				
Radiological studies				
Ultrasonography	As part of the initial evaluation of CKD, but also valuable in evaluating AKI. It is non-invasive, performed at the bedside and provides information regarding kidney size, echotexture, presence of cysts, kidney stones and possible obstruction. Doppler ultrasonography is employed to evaluate renal vascular flow to detect renal artery stenosis, renal vein thrombosis or kidney infarction, e.g., following kidney transplantation			
Computed tomography (CT)	CT with contrast may be used to evaluate kidney stones, complex kidney cysts, masses and to visualize the ureters. Contrast enhanced CT should be done in consultation with a radiologist to avoid unnecessary use of radiocontrast agents associated with AKI			
Magnetic resonance imaging (MRI)	It used in a variety of clinical settings and may be useful when ultrasound and CT are non-diagnostic and/or radiocontrast media cannot be administered due to allergy or decreased renal function. Nonetheless, it is important to note that use of gadolinium for MR angiography has been linked to nephrogenic systemic fibrosis (NSF) among patients with reduced renal function and consultation with a radiologist is recommended in this setting			
Renal arteriography	These are advanced forms of renal imaging which should be conducted for specific situations in consultation with a nephrologist			
Renal venography				
Voiding cystourethrography (VCUG)				
Radionucleotide studies				
Retrograde/anterograde pyelography

**Table 4.** Factors Affecting Biomarkers of Glomerular Filtration [16, 31–33].

Serum biomarker	Effect on the filtration marker	
	Increase	Decrease
SCr	Younger age	Older age
	Male sex	Female sex
	Large muscle mass	Protein restriction (renal disease, liver disease)
	Ingestion of cooked meat	Vegetarian diet
	Ketotic states, hyperglycemia	Muscle wasting (neuromuscular diseases, malnutrition)
	Drugs (cimetidine, trimethoprim, H2 histamine receptor antagonist)	Amputation
	Vigorous exercise	Hyperbilirubinemia
	Rhabdomyolysis	
SCysC	Older age	Female sex
	Male sex	Low body mass
	Large body mass	Immunosuppressive therapy (corticosteroids)
	Smoking status	Hypothyroidism
	States of inflammation	
	Hyperthyroidism	
BUN	Decreased effective circulating volume	Excessive volume expansion
	Increased dietary protein	Pregnancy
	Critical illness (fever, trauma, burns, sepsis)	Syndrome of inappropriate antidiuretic hormone
	Gastrointestinal bleeding	Dietary protein restriction
	Drugs (corticosteroids, tetracyclines)	Liver disease
Urine biomarkers		
Proteinuria	Defects in permselectivity of the glomerular filtration barrier to plasma proteins (e.g., glomerulonephritis or nephrotic syndrome) leading to glomerular proteinuria	
	incomplete tubular reabsorption of proteins (e.g., interstitial nephritis) leading to tubular proteinuria	
	increased plasma concentration of proteins (e.g., multiple myeloma, myoglobinuria) leading to overflow proteinuria	
	Urinary tract infection (UTI) or tumor	

## Causes of Drug-Induced Nephrotoxicity

Drug-induced nephrotoxicity may be the result of a combination of contributing factors including the Investigational Medicinal Product (IMP) mechanism of action (both on-target and off-target), the specific study population (e.g., in the elderly, patients with polycystic kidney disease), the presence of existing renal impairment at treatment initiation, individual genetic predisposition, and/or concomitant medications. It may manifest immediately following drug exposure (e.g., contrast agents), after days to weeks (e.g., non-steroidal anti-inflammatory drugs (NSAIDs), or aminoglycoside antibiotics), or only after extended exposure to the offending agent (e.g., phenacetin or cyclosporine), with high variability among agents and their nephrotoxic manifestations. As transient and modest degrees of DIN may remain asymptomatic and therefore undetected, renal function assessments should be planned to identify the early- and delayed-onset of DIN. Nephrotoxicity, manifesting as impaired renal function, may have different etiologies (Table 1).

CKD that has resulted from disease, drugs used in prior treatments, congenital renal abnormalities, or the natural aging process may hinder the kidneys’ ability to compensate for DIN, depending on baseline functional impairment/reserve. In general, drug classes have specific patterns of nephrotoxicity, which can be categorized by their effects on renal function (e.g., altered GFR due to angiotensin-converting enzyme (ACE) inhibition, diabetes insipidus, renal Fanconi syndrome, pseudo-hypo-aldosteronism), or structural abnormalities causing injury to specific areas in the kidney (e.g., the glomerulus, proximal or distal convoluted tubule). Tubulo-interstitial damage is the most common form of direct DIN. In contrast, idiosyncratic nephrotoxicity may present with a range of clinical syndromes including immune-mediated renal injury (e.g., gold therapy-induced membranous glomerulonephritis, drug-induced lupus-like syndrome, drug-induced glomerulosclerosis, protein deposits, calculi, infections secondary to immunosuppression).

DIN generally occurs more frequently in the presence of risk factors that increase patient vulnerability to nephrotoxicity. In particular, patients aged > 65 years or with pre-existing renal impairment regardless of age are at higher risk for DIN. Other concomitant diseases and conditions increase patient vulnerability to DIN, including medical conditions that cause renal hypoxia or decrease renal perfusion [10]. These factors need to be taken into consideration before allowing a high risk patient to be treated with a potentially nephrotoxic drug.

Several drugs and different contrast agents have the potential to cause or exacerbate renal injury and should be avoided during clinical trials (Table 5). If this is not possible, renal monitoring should be adapted to ensure patient safety. At the very minimum, all medications, including herbal supplements, should be documented in the concomitant medication list. Certain drugs may compete for renal tubular excretion, e.g., penicillin (acids), procainamide (bases), and all glucuronides, leading to drug interaction and potential toxicity. The risk of renal injury escalates in the presence of multiple risk factors. Patients with one or more risk factors (e.g., diabetes, cardiac failure, and myeloma) should be specifically monitored to identify the changes in renal function during long-term studies, in particular during dose escalation, when changes are made to co-medication, or during changes in patient volume status. This may include more frequent safety assessment in some or all of the patients in a trial.

**Table 5.** Drug Classes Commonly Associated with Nephrotoxicity or Altered Renal Physiology.

Drug class	Examples
Antihypertensives	Diuretics, ACE inhibitors^a^, ARBs^b^
Antimicrobials	Aminoglycoside antibiotics (gentamicin), sulphonamides, vancomycin, rifampicin, amphotericin B
Immunotherapy	Tacrolimus, cyclosporine, cyclophosphamide, immune checkpoint inhibitor Interferon-alpha therapy, Interleukin-2 therapy, chimeric antigen receptor T (CAR-T)
Pain treatment	NSAIDs^c^
Heavy metals	Gold
Antineoplastic agents	Cisplatin, gemcitabine, doxorubicin, ifosfamide, methotrexate
Antiviral therapies	Tenofovir, acyclovir, adefovir, indinavir
Others	Contrast media, lithium, deferasirox, herbal, and natural products (e.g., Aristolochia, St. John’s wort), tyrosine kinase inhibitor

Only the most common nephrotoxic agents are listed

^a^*ACE* *inhibitors* Angiotensin-converting enzyme inhibitors

^b^*ARBs* Angiotensin receptor blockers

^c^*NSAIDs* non-steroidal anti-inflammatory drugs

## Assessing Drug Potential for Nephrotoxicity

### Preclinical Evaluation and Biomarkers

During preclinical drug safety evaluation, nephrotoxicity is generally assessed in vivo*,* while in vitro evaluation is used to explore renal drug transport and metabolism and to study the mechanism of DIN. Renal toxicity of an IMP can manifest as a functional change, as overt renal lesions, or as injury associated with histopathological changes, frequently depending on the dose and duration of exposure. The effect may be regional or may be diffused and affected several regions, resulting in specific clinical manifestations, urinalysis changes, and/or biomarker patterns. Histopathological signs of DIN include the following:

- Glomerular podocyte foot process effacement
- Glomerular sclerosis
- Glomerular epithelial crescent formation
- Acute tubular necrosis, vacuolization, obstruction
- Interstitial inflammation
- Tubular atrophy
- Tubulitis
- Nephrocalcinosis
- Renal papillary necrosis
- Renal artery or renal vein hypertrophy, anatomical change, dilation, or rupture
- Renal vasculitis or thrombosis, or vascular occlusion or vascular obliteration
- Renal intravascular/perivascular inflammatory changes/infiltrates
- Urinary calculi
- Crystal deposition

Other preclinical findings that may indicate nephrotoxicity include dose-dependent increases in serum creatinine, microscopic or gross hematuria, proteinuria, metabolic acidosis, or urinary crystal formation/excretion. Monitoring of biomarkers that allow early identification of the onset and severity of DIN and that track the reversal of DIN after drug withdrawal may additionally provide mechanistic insight into IMP-related renal toxicity. As a result, novel preclinical biomarkers of renal toxicity have been developed (Table 6).

**Table 6.** Preclinical Urinary Biomarkers for Renal Toxicity.

Biomarker	Used to detect	Validated for preclinical use by	Provides additional and complementary information to BUN and SCr	Qualified for Clinical use
Kidney Injury Molecule-1 (KIM-1)	Acute drug-induced kidney tubular alterations	PSTC^a^	Yes	Qualified by FDA for clinical use as follows: A safety composite biomarker panel may be used in conjunction with traditional measures to aid in the detection of kidney tubular injury in phase 1 trials in healthy volunteers when there is an a priori concern that a drug may cause renal tubular injury in humans The composite measure is not intended to replace standard measures (such as serum creatinine, BUN, urinalysis, urine albumin, and urine total protein) used to monitor for drug-induced renal injury in clinical trials, but is intended to be used in addition to these standard measures In addition, other biomarkers are currently under evaluation*
Albumin				
Total protein	Acute drug-induced glomerular alterations/damage and/or impairment of kidney tubular reabsorption			
Cystatin C				
β2-microglobulin				
Clusterin	Acute drug-induced kidney tubular alterations			
Trefoil Factor-3				
Urinary Renal Papillary Antigen-1 (RPA-1)	acute drug-induced renal tubule alterations, particularly in the collecting duct in male rats	ISLI/HESI^b^		

*Biomarkers currently under evaluation: interleukin-18 (IL-18), Neutrophil Gelatinase-Associated Lipocalin (NGAL), Netrin-1, Fatty Acid-Binding Proteins (FABP), urinary exosomes, Tissue Inhibitor of Metalloproteinases 2 (TIMP2) and Insulin Like Growth Factor Binding Protein 7 (IGFBP7) [24]

^a^*PSTC* Predictive Safety and Testing Consortium

^b^*ILSI* International Life Sciences Institute, *HESI* Health and Environmental Sciences Institute

Due to variability and multi-dimensional urine composition in human clinical setting, interpretation of urinary kidney biomarkers evaluated in preclinical models will profit from additional clinical assessments in humans. Urinary creatinine should be measured to normalize (creatinine-index) biomarker concentrations and assessment of sensitivity and specificity of each biomarker is important during transitioning to clinical use.

### Clinical Evaluation

Assessing an IMP’s potential to cause nephrotoxicity must consider the following factors:

- The IMP-specific preclinical or early clinical signals of nephrotoxicity
- The clinical predictive value of animal models, at relevant exposure levels
- Pre-existing conditions in the patient population that may predispose to DIN
- Interaction with concomitant medications
- Renal effects of other IMPs in the same drug class

When transitioning from preclinical to clinical development, the first step is to determine the IMP’s potential to cause nephrotoxicity. Based on our drug development experience, we have developed classification nomenclature for IMPs, namely DIN-L1 (Level 1) if there is no known potential to cause DIN, or DIN-L2 (Level 2) if there is evidence of or potential for DIN (Table 7). As DIN may be caused by indirect mechanisms or manifest only after longer clinical exposure, it is recommended that DIN-L1 IMPs with a new mechanism of action (MoA) that is not well characterized or are planned to be used in patients with pre-existing nephropathy, are conservatively classified as DIN-L2 during early phase clinical studies.

**Table 7:** Compound Potential of Renal Dysfunction.

DIN-L1 compound (If ALL of the following criteria are met)	DIN-L2 compound (If ANY of the following criteria are met)
No adverse preclinical in vitro or in vivo renal safety signals	Positive preclinical in vitro or in vivo renal safety signal: renal function deterioration, histopathology, proteinuria
MoA^a^ well characterized with no known nephrotoxicity, and MoA does not affect the kidney, glomerular filtration rate, tubular handling or protein excretion	New MoA that is not well characterized, or MoA with renal effects, e.g., changes in filtration, protein, glucose, water or electrolyte handling
No renal safety findings associated with compound or drug class	Known renal safety findings associated with compound or drug class

Criteria are irrespective of study phase

^a^*MoA* mechanism of action

Even though a compound may initially be classified as either DIN-L1 or DIN-L2, reassessment of renal effects should occur after Phase 2 and Phase 3 studies. Appropriate renal monitoring parameters should be included in respective clinical trial protocols. An IMP’s potential to cause DIN should be reassessed regularly during the development lifecycle, at clinical trial phase transition, and when relevant safety data become known, considering all available preclinical and clinical evidence (i.e., laboratory values, adverse event profile, biomarkers), and internal or external safety data from similar drugs. Should DIN manifest in a DIN-L1 IMP in a specific patient population (e.g., the elderly, pediatrics, first in human or in patients with diabetes, cardiac failure, or chronic kidney disease), a reassessment should be conducted based on available data to determine if the IMP should be reclassified to DIN-L2 and the monitoring plan updated. Early studies with DIN-L2 IMPs may exclude subjects > 65 years, subjects with certain concomitant diseases predisposing to renal impairment (e.g., hepatic or cardiac failure), and the use of potentially nephrotoxic co-medication until the renal safety of the IMP has been established.

In the absence of any clinical findings of DIN during early phase clinical studies, a DIN-L2 IMP can be reclassified to DIN-L1 after careful consideration (note that DIN may only manifest after 6 to 12 months’ treatment). Drug development teams are advised to include renal safety monitoring in long-term studies in the target population if applicable (may not be applicable in limited drug exposure, short-term treatment, or in oncology, where other criteria may apply), and in consultation with renal experts and relevant safety board(s). The IMP’s potential to cause nephrotoxicity will impact the choice of appropriate level of safety monitoring, the clinical trial eligibility criteria and discontinuation criteria, and the selection of specific renal safety markers to be measured/monitored.

### Renal Monitoring During the Clinical Development Program

Proactive monitoring for potential nephrotoxicity allows for timely detection and enhances patient safety throughout the clinical program. A monitoring program consists of selected renal safety assessments performed with the appropriate assessment frequency. Depending on whether the IMP is DIN-L1 or DIN-L2, the development phase, and the target patient population risk profile, monitoring requirements will vary from Base to Observation to Intense Monitoring (Table 8). Monitoring may need to be increased from Base to Observation or Intense for any of the following situations:

- Inclusion of a new at-risk patient population
- Addition of concomitant nephrotoxic medication, or
- Identification of a potential renal safety signal

**Table 8.** Monitoring and Assessment of Renal Event During Drug Development.

Monitoring requirement	Recommendation	Assessments	Assessment Frequency
Base	for DIN-L1 compounds during Phase 2 and 3 in the absence of a population-related liability for DIN	Serum Creatinine, BUN, Electrolytes (Na, Ca, K, Cl, HCO3) Urine^c^ Dipstick (Spot urine sample)^d^	Single baseline assessment^a^
			Steady State assessment
			6-month intervals during study
			Final assessment at last follow-up visit (at least > 48 h after last dose, depending on PK profile of the study treatment)
Observation	for DIN-L1 compounds during Phase 1 for DIN-L2 compounds during Phase 3		Baseline assessments^a^
			Steady state assessment
			Monthly during study for the first 4 months, and then every 3 months
			Final assessment at last follow-up visit (at least > 48 h after last dose, depending on PK profile of the study treatment)
Intense	for DIN-L2 compounds during Phase 1, and 2 dose-range studies (early clinical studies)^b^		Two baseline assessments^a^
			24 to 48 h after 1st and 2nd dosing days
			Every 2 weeks for the first 3 months, monthly up to 12 months, and then every 3 months, depending on the overall study duration^e^
			Final assessment at last follow-up visit (at least > 48 h after last dose, depending on PK profile of the study treatment)

*DIN* drug-induced nephrotoxicity, *BUN* blood urea nitrogen, *PK* pharmacokinetics

^a^Includes a screening/pre-dose and a baseline assessment, and specific renal biomarkers where appropriate

^b^During Phase 3 and late-phase studies, assessment frequency may be adapted (e.g., Intense Monitoring may be performed in selected patient groups with Observation Monitoring in the remaining groups), depending on the accumulated drug safety profile, and the profile of DIN for DIN-L2 compounds during earlier studies

^c^For Observation/ Intense monitoring, urinary biomarkers are preferable; however, in some cases, plasma markers are required

^d^Any positive dipstick finding should be followed up by microscopy if the sample is adequately preserved and timing is same day; otherwise, another fresh sample needs to be obtained as soon as possible. If a central lab is used, an appropriate transport medium to prevent RBC lysis is needed

^e^Visit frequency should be adapted depending on the accumulated drug safety profile and the profile of DIN during earlier phase studies. For special situations (e.g., in oncology), or to accommodate the compound PK profile or protocol visit schedule, the assessment frequency might need to be adjusted

The collection of pharmacokinetic (PK) samples is recommended, especially for DIN-L2 IMPs, or when renal events occur, to determine whether nephrotoxicity correlates with drug exposure levels. PK sampling should be performed at steady state at regular intervals depending on the DIN monitoring level. PK sampling is also recommended following dose adjustment, or in the presence of other risk factors that may increase the risk for nephrotoxicity. Some PK parameters to be considered should include CMax, AUC, elimination half-life, protein binding or association with albumin or bilirubin (BR), PkA, the use of parametric analysis, or population pharmacokinetics (popPK) models stratified for CrCL (creatinine clearance). Health authority guidelines should be referenced to determine the most applicable parameters.

The collection and storage of additional plasma and urinary samples in early phase clinical studies that will allow the team to evaluate genetics and biomarkers at a later time for DIN-L2 IMPs in consultation with the relevant experts is recommended. These samples may be evaluated during the study to monitor patient safety, at study completion, or at a later stage as required. For DIN-L2 IMPs in particular, prospectively collected urine samples may become important if companion diagnostic tests for IMP nephrotoxicity have been or will be developed and validated.

Additional laboratory and clinical assessment (e.g.; evaluation of the patient hemodynamic status) may provide information regarding specific aspects of kidney function, for example, renal causes for electrolyte or water imbalances. Study teams should be mindful of the storage requirements for certain tests (e.g., alkaline urine needed to prevent B2-microglobulin degradation or required centrifugation for certain assays).

### Considerations for Clinical Trial Design and Protocols

As noted above, study eligibility criteria and monitoring will be determined by the IMP level (DIN-L1 or DIN-L2), development phase, and the presence of any population-related risk factors of nephrotoxicity, including concomitant medications, the underlying disease, the current CKD stage, and comorbid conditions. Eligibility criteria and clinical assessments should follow a company’s guidance for clinical trial protocols.

During protocol and assessment schedule creation, the IMP’s monitoring level should be applied to select the appropriate tests and measurement frequency. In addition, if any renal adverse event is reported, additional testing, including urinalysis and blood testing, should be performed.

## Definition of Renal Event

A renal event is defined as abnormal clinical signs and/or symptoms and/or laboratory abnormalities that reflect impaired renal function, or a confirmed change in urine composition such as the presence of protein, glucose, or blood.

While the most sensitive diagnosis of a renal event is a confirmed increase in serum creatinine of ≥ 25% compared to baseline (corresponding to a decrease of eGFR by approx. 20%), eGFR is preferred in evaluating for renal events as transient increases in serum creatinine may occur as a result of non-renal factors, such as changes in hydration status, diet or exercise. Therefore, confirmation of an event with a second assessment is required to ensure a renal etiology of the event. Since creatinine is often measured frequently, it should be diligently evaluated as well. An increase of 25–50% in serum creatinine even in normal ranges may not necessarily qualify as an adverse event, but should be evaluated to better clarify the occurrence.

In the presence of low baseline serum creatinine values, such as those with low muscle mass (e.g., pediatric population or cancer patients), detecting a 25% change from baseline may be limited by assay sensitivity. Similarly, changes in muscle mass will affect creatinine clearance; decreasing muscle mass will impede the ability to identify a renal event, while increased muscle mass or muscle breakdown may increase creatinine levels and trigger false events. If muscle mass changes during the course of the study, it is advised to define renal events by changes in eGFR rather than serum creatinine changes.

Any positive dipstick finding should be followed up by microscopy if the sample is adequately preserved and timing is same day; otherwise, another fresh sample needs to be obtained as soon as possible. Urine protein present on a dipstick should be quantified using protein:creatinine ratio (PCR) measurement.

### Management and Follow-Up

Upon diagnosis and confirmation of a renal event, some general procedures, and some event specific activities, as illustrated in Table 9, are indicated depending on the severity of the event and the clinical status of the patient. Whenever a renal event is identified, a detailed patient history and examination, including the parameters below are indicated to identify and treat the patient:

- Blood pressure
- Signs and symptoms such as fever, headache, shortness of breath, cardiac murmur, back or abdominal pain, hepatomegaly, dysuria or hematuria, edema
- Changes in body weight, fluid intake, voiding pattern, or urine output
- Concomitant events such as trauma, surgical procedures, cardiac or hepatic failure, nephrotoxin administration, or other diseases or causes, e.g., dehydration, tumor lysis.

**Table 9.** Specific Renal Alert Criteria, Actions, and Follow-Up.

Renal event	Actions
eGFR decrease 25–49%	Consider causes and possible interventions
	Repeat laboratory values within 48 h of receipt of abnormal test results. Assess patient for signs and symptoms of illness, AKI, etc
eGFR decrease ≥ 50%^a^ OR if < 18 years old, eGFR < 35 mL/min/1.73 m^2^	Consider causes and possible interventions
	Repeat assessment within 24–48 h if possible
	Repeat laboratory values within 48 h of receipt of abnormal test results. Assess patient for signs and symptoms of illness, AKI, etc
	Consider drug interruption or discontinuation unless other causes are diagnosed and corrected
	Consider referral to nephrologist for diagnosis and management
	Consider patient hospitalization and specialized treatment
New onset dipstick proteinuria ≥ 3 + OR PCR ≥ 1 g/g Cr	Confirm presence of true proteinuria by quantification. (protein: creatinine on first morning void)
	Consider causes and possible interventions
	Assess serum albumin & serum total protein
	Repeat assessment to confirm
	Consider drug interruption or discontinuation unless other causes are diagnosed and corrected
	Consider referral to a nephrologist
New onset hematuria ≥ 3 + on urine dipstick	Obtain urine microscopy to distinguish hemoglobinuria or myoglobinuria from hematuria
	Assess sCr
	Exclude infection, trauma, calculi, bleeding from the distal urinary tract/bladder, menstruation
	Consider bleeding disorder
Follow-up of renal events	
Monitor patient regularly (frequency dependent on clinical course and consultant advisement) until:	
Event resolution: sCr within 10% of baseline or PCR < 1 g/g Cr, or ACR < 300 mg/g Cr, or	
Event stabilization: sCr level with ± 10% variability over last 6 months or PCR stabilization at a new level with ± 50% variability over last 6 months	
Analysis of urine renal markers in samples collected over the course of the DIN event	

*eGFR* estimated glomerular filtration rate, *AKI* acute kidney injury, *sCR* serum creatinine, *PCR* protein-creatinine ratio, *ACR* albumin-to-creatinine ratio, *DIN* drug-induced nephrotoxicity

^a^As per KDIGO 2012 criteria for AKI

## Special Populations

### Pediatrics

Clinical trials in children are usually performed after the adult population has been studied; however, they may be conducted first or simulataneously with adults if the disease state primarily or frequently occurs in children. In either scenario, pediatric studies require additional consideration. In general, renal function (eGFR) in the child is considered to be comparable to the adult after two years of age. However, muscle mass, and therefore serum creatinine and measured GFR should be corrected for body surface area which increases with increasing age reaching adult values when growth during the second decade of life.

Disorders increasing the risk of CKD, and DIN in children and adolescents include the following:

- Family history of genetic kidney disease
- Low birth weight
- Teratogens: ACE inibitors, Angiotensin receptor blockers (ARBs), congenital infections, NSAIDs
- Underlying renal disease including renal dysplasia/hypoplasia
- Urologic disorders—especially obstructive uropathies
- History of perinatal hypoxemia, maternal oligohydramnios, or other acute renal insult

Renal events in the pediatric population should be defined as 25% decrease in eGFR [25] using age-specific normal values for eGFR and serum/urinary normal values. AKI can best be assessed using KDIGO criteria [26]. The Schwartz age-specific eGFR formula [23] should be used to calculate eGFR in children. Since the most common cause of secondary hypertension in children is renal disease, blood pressure should be monitored in accordance with the Clinical Practice Guideline for Screening and Management of High Blood Pressure in Children and Adolescents [27]. Proteinuria may be seen in children in a benign condition called benign orthostatic (postural) proteinuria, which is not associated with renal dysfunction and is considered a normal physiologic variant. Thus, whenever assessing a positive dipstick for proteinuria in children, a first morning sample should be used to eliminate this potentially complicating variable.

### Cancer Patients

Chronic kidney disease is prevalent in cancer patients regardless of tumor type [28, 29]. Disease progression and compromised renal function in cancer patients resulting in decreased clearance rates may lead to higher drug exposure. In addition, cancer patients are frequently exposed to potentially nephrotoxic chemotherapeutic agents (i.e., cisplatin).

### Prediction of DIN in Cancer Patients and Oncology IMP Classification

Therapeutic drug exposure is an important determinant of DIN in oncology patients and may vary significantly between treatment regimens. Due to higher levels of drug exposure during preclinical evaluation, preclinical renal toxicity signals may not translate into clinical nephrotoxicity. In other words, even though an IMP could be classified as DIN-L2 based on preclinical toxicity findings at high exposure levels, DIN may not occur in patients at lower exposure levels. In addition, modern molecular targeted oncology therapeutic IMPs (e.g., tyrosine kinase inhibitors) generally lack tissue specificity and selectivity and may directly or indirectly affect multiple organs, including the renal system [30]. Therefore, unless a specific mechanism for potential renal toxicity can be identified or suspected, DIN risks may be difficult to predict.

In IMPs whose drug exposures inducing preclinical DIN are unlikely to be achieved in humans; and provided a sufficient safety margin has been established when assessing the therapeutic drug concentrations, DIN-L1 monitoring may be appropriate. Such a decision will be based on the early safety data in the context of predicted therapeutic use, consideration of factors that may increase drug exposure (e.g., hepatic or renal impairment), feasible risk mitigation strategy, and on DIN potential in the target population. For IMPs assigned to DIN-L2 prior to human testing, a re-classification to L1 may be considered on the basis of a conservative risk assessment of the preclinical evidence, if there is adequate evidence to determine that no clinical or sub-clinical renal toxicity signal is identified at the completion of the Phase I oncology trial(s).

### Mitigation Strategies for DIN in Cancer Patients

Pre-existing renal impairment is a risk factor for DIN, where the risk of drug-induced renal toxicity is proportional to the deterioration of renal function in cancer patients. Therefore, caution should be exercised in treating cancer patients with existing renal impairment, especially for DIN-L2 IMPs. In addition, adequate hydration remains fundamental for all patients receiving IMPs with nephrotoxic potential. Changes in renal function during and after drug administration should be correlated with drug exposure levels, and dose interruption or reduction is recommended to mitigate the risk of potential renal toxicity. The renal function monitoring frequency may also need to be adapted based on the accumulated renal safety information for the IMP and indication. In specific situations where the accurate assessment is imperative to know an actual GFR to determine accurate dosing or in situations where even small changes in GFR are significant, GFR should be measured using radionucleotide techniques (e.g., iohexol) as these are more precise than creatinine or eGFR (Table 3).

## Investigational Medicinal Product discontinuation

The decision to discontinue the IMP, temporarily or permanently, in any individual patient is made by the investigator based on patient safety and the risk–benefit profile of the treatment. From a renal vantage point,

- Consider discontinuing or interrupting study treatment for a patient if individual eGFR decreases ≥ 50% compared to baseline (and is considered clinically significant), or in the event of treatment-emergent quantified proteinuria (ACR > 1000 mg/g or > 100 mg/mmol; PCR ≥ 2 g/g or > 200 mg/mmol), unless the event is deemed not drug related, related to natural disease progression, or if the benefit/risk assessment supports continuing treatment.
- A renal event leading to patient discontinuation should be followed up until event resolution (Serum Cr within 10% of baseline, PCR < 1 g/g Cr, ACR < 300 mg/g Cr) or stabilizes.

DIN-related modifications to the trial protocol should be based on IMP level (DIN-L1 or DIN-L2), the known safety profile of the IMP, and the overall risk–benefit profile. Guidance from relevant experts (e.g., nephrologists) is recommended for DIN-L2 or DIN-L1 IMPs in a patient population with an increased risk for DIN.

## Conclusions

Given that DIN may influence the choice as to whether or not a specific therapy should be prescribed, the benefit-risk of the compound should be considered to determine whether the disease for which a drug is being developed is severe enough that some degree of kidney damage may be acceptable, particularly for drugs where changes in kidney function are mechanism-dependent and no alternative therapy is available. If reversibility of kidney damage is indeed established, continued dosing of the IMP could be considered, but with more frequent safety assessments to determine if kidney function is stabilizing or further deteriorating. If deterioration of renal function is observed, the drug should be discontinued. DIN in drug development should be anticipated through preclinical studies considering validated biomarkers and by identifying risk factors. The clinical trial team should focus on identification of subjects at risk of DIN, and the development of clear protocols to proactively define the necessary steps to reduce or eliminate this risk for patients enrolled in clinical trials.

Current definitions of renal injury are based on changes in SCr that relate to changes in GFR and not to renal injury itself. These classifications need to be refined for DIN, or a new classification may be needed explicitly for DIN. Another disadvantage is that changes in SCr occur not earlier than 24 to 48 h after kidney damage, which delays the diagnosis of DIN, increasing the risk of irreversible kidney damage. A major obstacle to earlier diagnosis of kidney damage, irrespective of etiology, is a lack of validated biomarkers to predict damage to the kidneys holistically as well as different nephron segments. Intensive research and collaboration between academia, the pharmaceutical industry, and health authorities are currently focused on the development and qualification of renal safety biomarkers. Those identified by the Nephrotoxicity Working Group of the PSTC have received approval from the Food and Drug Administration and European Medicines Agency for use in preclinical and clinical Phase I studies. It is expected that their validation and integration into different phases of clinical drug development will facilitate the early detection of DIN, leading to the development of appropriate risk mitigation and minimization strategies. Developing more sensitive methods to predict DIN in preclinical studies and early clinical detection of kidney damage would help ensure patient safety and facilitate informed decisions during drug development.
